# SCF/c-Kit-activated signaling and angiogenesis require Gαi1 and Gαi3

**DOI:** 10.7150/ijbs.82855

**Published:** 2023-03-27

**Authors:** Hua-jian Shan, Kun Jiang, Ming-zhi Zhao, Wen-jing Deng, Wen-hao Cao, Jia-jun Li, Ke-ran Li, Chang She, Wei-feng Luo, Jin Yao, Xiao-zhong Zhou, Dan Zhang, Cong Cao

**Affiliations:** 1 Clinical Research Center of Neurological Disease, The Second Affiliated Hospital of Soochow University, Institution of Neuroscience, Soochow University, Suzhou, China.; 2 The Affiliated Eye Hospital, Nanjing Medical University, Nanjing, China.; 3 Department of Orthopedics, The Second Affiliated Hospital of Soochow University, Suzhou, China; 4 Vascular Surgery Department, Kunshan Hospital of Traditional Chinese Medicine, Kunshan, China.; 5 Department of Otorhinolaryngology, The First Affiliated Hospital of Soochow University, Suzhou, China.

## Abstract

The stem cell factor (SCF) binds to c-Kit in endothelial cells, thus activating downstream signaling and angiogenesis. Herein, we examined the role of G protein subunit alpha inhibitory (Gαi) proteins in this process. In MEFs and HUVECs, Gαi1/3 was associated with SCF-activated c-Kit, promoting c-Kit endocytosis, and binding of key adaptor proteins, subsequently transducing downstream signaling. SCF-induced Akt-mTOR and Erk activation was robustly attenuated by Gαi1/3 silencing or knockout (KO), or due to dominant negative mutations but was strengthened substantially following ectopic overexpression of Gαi1/3. SCF-induced HUVEC proliferation, migration, and capillary tube formation were suppressed after Gαi1/3 silencing or KO, or due to dominant negative mutations. *In vivo*, endothelial knockdown of Gαi1/3 by intravitreous injection of endothelial-specific shRNA adeno-associated virus (AAV) potently reduced SCF-induced signaling and retinal angiogenesis in mice. Moreover, mRNA and protein expressions of *SCF* increased significantly in the retinal tissues of streptozotocin-induced diabetic retinopathy (DR) mice. SCF silencing, through intravitreous injection of SCF shRNA AAV, inhibited pathological retinal angiogenesis and degeneration of retinal ganglion cells in DR mice. Finally, the expression of SCF and c-Kit increased in proliferative retinal tissues of human patients with proliferative DR. Taken together, Gαi1/3 mediate SCF/c-Kit-activated signaling and angiogenesis.

## Introduction

Vascular dysfunction is a major pathogenesis in several human diseases, including heart failure, stroke, diabetes, and retinal vascular diseases 1-3. It results in a shortage of nutrients and oxygen, which leads to metabolic injuries, cell damage, and even organ failure 4, 5. Resting-state endothelial cells rarely proliferate and are at the lumen of blood vessels 4, 5. Various growth factors, including VEGF as well as other stimuli (like hypoxia), promote endothelial cell proliferation, sprout elongation, and lumen formation 6-8. Tip cells of adjacent sprouts then join to form new vessels 6-8. This process is termed angiogenesis 4, 5, 8-10.

Stem cell factor (SCF) binds and activates c-Kit, a receptor tyrosine kinase (RTK) 11, 12. It is a dimeric molecule regulating several key cellular behaviors, including cell survival, migration, and proliferation, as well as physiological functions, including hematopoiesis, melanogenesis, and gametogenesis 12. SCF binding to c-Kit induces its autophosphorylation 13-15. Key adaptor proteins, including Grb2, Gab2, Shc, and Sos, thereafter bind to the intracellular region of c-Kit 11, 16. Subsequently, multiple downstream signaling cascades (PI3K-Akt-mTOR, Erk and others), are activated 11, 16, 17. Elucidating the mechanism of SCF/c-Kit signaling transduction is, therefore, of great significance.

SCF is important for angiogenesis. Matsui *et al.* reported that SCF treatment in endothelial cells could activate pro-angiogenic reactions and enhance mobility and the formation of capillary tubes in endothelial cells 18. Fang *et al.* reported that c-Kit deficiency hindered vascular endothelial stem cell proliferation and blocked angiogenesis *in vivo* 19. Wang *et al.* reported that the activation of c-Kit by SCF could promote survival and suppress apoptosis in vascular smooth muscle cells 20. SCF activates c-Kit signaling and is important for the formation of hematopoietic stem cells 17. Herein, we examined the role of G protein subunit alpha inhibitory (Gαi) proteins in SCF-activated signaling and angiogenesis.

There are three primary members of Gαi proteins, namely Gαi1, Gαi2, and Gαi3. These associate with Gαi protein-coupled receptors (GPCRs) and inhibit adenylyl cyclase (AC) 21, 22. Studies have established the role of Gαi proteins in transducing signals for various RTKs 23-32 and also a few non-RTK receptors 33, 34. Epidermal growth factor (EGF)-stimulated EGFR associated with Gαi1 and Gαi3 mediates the adaptor protein Gab1 association and activates the downstream Akt-mTOR 31, 32.

Moreover, Gαi1 and Gαi3 are both indispensable for mediating the activation of Akt-mTOR cascade by KGF 30 and BDNF 28. Gαi1 and Gαi3 can bind to ligand-activated KGFR 30 and TrkB (the BDNF receptor) 28 and transduce downstream signals for their activation. Interleukin 4 (IL-4)-induced signaling activation also requires Gαi1 and Gαi3 28. Specifically, with IL-4 stimulation, Gαi1/3 associated with IL-4Rα's intracellular domain, promotes IL-4Rα's endocytosis, endosomal translocation, and Akt-mTOR activation 33.

Our group has recently explored Gαi proteins' role in angiogenesis. Following VEGF stimulation, Gαi1 and Gαi3 can promote VEGFR2's endocytosis and downstream cascade activation 27. VEGF-induced pro-angiogenic activity was prevented by Gαi1 and Gαi3 silencing or knockout (KO) 27. Phosphoenolpyruvate carboxykinase 1's (PCK1) association with Gαi3's transcription factor, GATA binding protein 4 (GATA4) increases the transcription and expression of Gαi3 in endothelial cells and promotes angiogenesis 23. In response to R-spondin3 (RSPO3) stimulation, Gαi1/3 associated with LGR4 (RSPO3 receptor) and the adaptor protein, Gab1, thus transducing downstream Akt-mTOR signaling and promoting angiogenesis 34. Herein, we evaluate the possible role and underlying mechanism of Gαi1/3 action in SCF/c-Kit-activated signaling and angiogenesis.

## Results

### Double KO of Gαi1 and Gαi3 in mouse embryonic fibroblasts (MEFs) abolishes SCF-induced signaling

**Figure 1:**
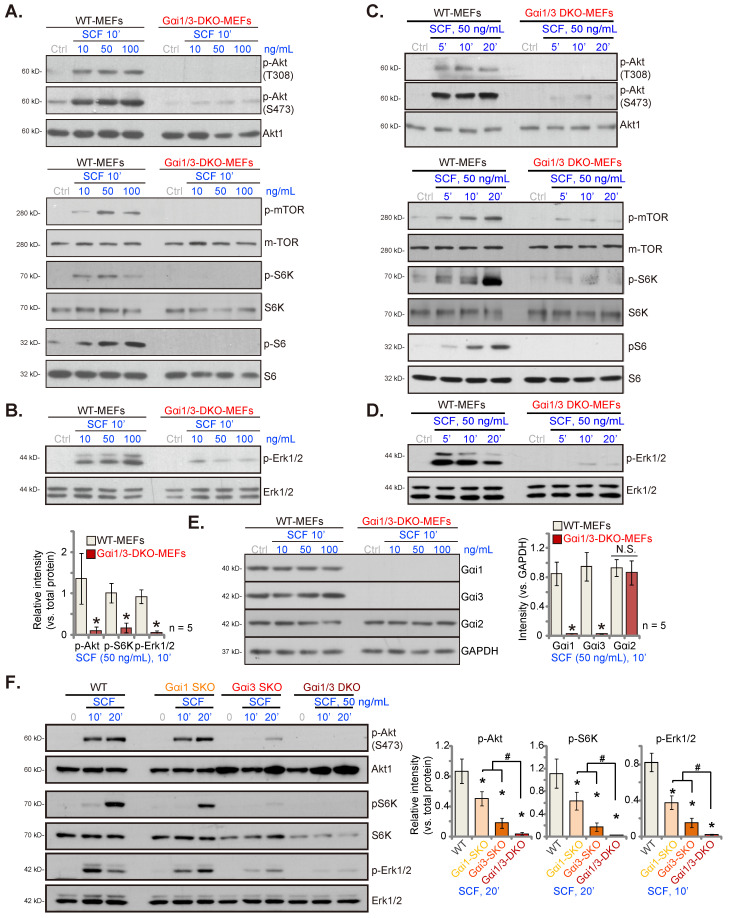
Double knockout of Gαi1 and Gαi3 in mouse embryonic fibroblasts (MEFs) abolishes SCF-induced signaling. The listed MEFs were stimulated with SCF at indicated concentrations and cultured for indicated periods, and the listed signaling protein levels were examined (**A**-**F**). “Ctrl” refers to PBS treatment. *P < 0.05 versus “WT MEFs”. **^#^*P*** < 0.05 (**F**). “N. S.” denotes ***P*** > 0.05.

Wild type (WT) MEFs and Gαi1 plus Gα3 double KO (DKO) MEFs were used to investigate Gαi proteins' involvement in SCF-induced signaling, as reported previously 26-28, 30, 31, 33, 34. These cells were treated with SCF (at 10, 50, or 100 ng/mL). After 10 min, the levels of different signaling proteins were examined. In WT MEFs, SCF robustly increased the phosphorylation of Akt, mTOR (Ser-2448), S6K (Thr-308), and S6 (Ser-235/236), all indicating activation of the Akt-mTOR cascade (Figure **1A**). However, in Gαi1/3-DKO MEFs, SCF-activated Akt-mTOR was blocked at all tested concentrations (10, 50, or 100 ng/mL) (Figure **1A**). Moreover, SCF induced Erk activation by inducing Erk1/2 phosphorylation in WT MEFs (Figure **1B**), which was suppressed in the DKO MEFs (Figure **1B**). Among the tested concentrations, 50 ng/mL of SCF caused robust signaling activation in MEFs (Figures **1A** and **B**).

Figure **1C** showed that treatment with SCF (50 ng/mL) activated the Akt-mTOR cascade and increased phosphorylation of Akt, mTOR, S6K, and S6 in WT MEFs in a time-dependent manner. Moreover in WT MEFs, SCF-induced Erk1/2 phosphorylation (50 ng/mL) was observed within 5 min after treatment and lasted for at least 20 min (Figure **1D**). Remarkably, SCF-induced Akt-mTOR (50 ng/mL) and Erk activation in MEFs was nullified by Gαi1/3-DKO (Figures **1C** and **D**). Total Akt, mTOR, S6K, S6, and Erk1/2 levels were equivalent in these MEFs (Figures **1A**-**D**). The blot data in Figure **1E** confirm Gαi1 and Gα3 depletion in the DKO MEFs, with an intact expression of the Gα2 protein (Figure **1E**). SCF (10, 50, or 100 ng/mL for 10 min) did not alter the expression of Gαi proteins in WT MEFs (Figure **1E**).

The assessment of the individual role of Gαi1 or Gα3 in SCF-induced signaling showed that Gαi1 single KO (SKO) or Gαi3 SKO in MEFs resulted only in partial reduction of Akt (Ser-473), S6K, and Erk1/2 phosphorylation by SCF (50 ng/mL) (Figure **1F)**. Only in Gαi1/3-DKO MEFs, SCF-activated signaling was completely blocked (Figure **1F**).

To silence Gαi1 and Gαi3, the Gαi1 (murine) shRNA-packed lentiviral particles and the Gαi3 (murine) shRNA-packed lentiviral particles, were constructed as reported previously 26-28, 30, 34. These were co-transfected into WT MEFs. After selection (by puromycin), stable lines were obtained, namely “shGαi1/3” MEFs. Control MEFs were stably transduced with lentiviral scramble non-sense shRNA control (“shC”). SCF (50 ng/mL) induced robust activation of Akt-mTOR and Erk and increased Akt (Ser-473), S6, and Erk1/2 phosphorylation in shC MEFs (Figure **S1A**), which was dramatically inhibited in shGαi1/3 MEFs (Figure **S1A**). The downregulation of Gαi1 and Gα3 in the shGαi1/3 MEFs with no change in the levels of Gαi2 protein was verified (Figure **S1B**).

### SCF-induced membrane c-Kit internalization in MEFs requires Gαi1 and Gαi3

Following SCF stimulation, c-Kit associates with several key adaptor proteins, including Grb2, Gab2, and Shc, and results in subsequent phosphorylation of Gab2 and Shc to promote downstream signaling 11. We discovered that Gαi1/3 could associate with ligand-activated receptors (IL-4Rα, TrkB, VEGFR2, etc.), thus promoting receptor internalization and endocytosis and transducing downstream signals 27, 28, 33, 35. Herein we showed that SCF-activated c-Kit underwent membrane internalization (Figure **S2A**). Cell membrane-localized c-Kit protein levels decreased remarkably in WT MEFs after SCF treatment (Figure **S2A**). The membrane c-Kit internalization was fast and started within 1 min of SCF treatment (Figure **S2A**), and by 5 min, the majority of membrane c-Kit protein was internalized (Figure **S2A**). Gαi1 and Gαi3 were required for SCF-induced c-Kit internalization, as membrane c-Kit internalization was prevented by Gαi1/3 DKO in MEFs (Figure **S2B**). Total c-Kit protein levels were unchanged following SCF treatment in MEFs (Figure **S2B**).

### Dominant negative (DN) mutants of Gαi1 and Gαi3 disrupt SCF-induced c-Kit internalization and binding of adaptor proteins and prevent activation of downstream signaling

**Figure 2:**
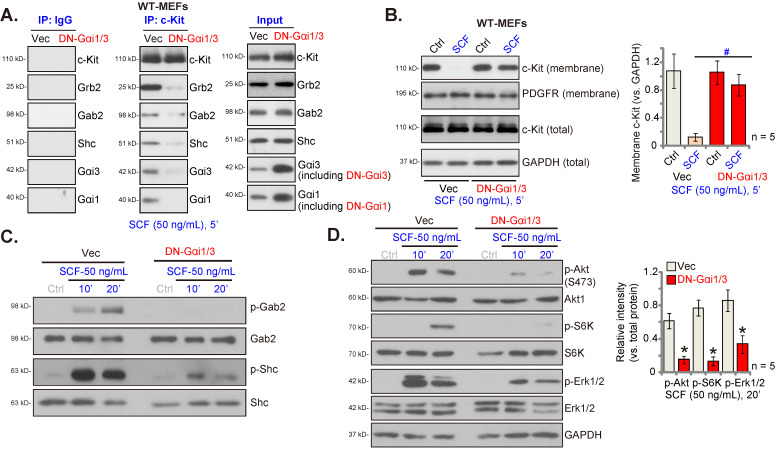
Gαi1 and Gαi3 dominant negative (DN) mutants disrupt SCF-induced c-Kit internalization and binding of adaptor proteins and prevent downstream signaling activation. Stable WT MEFs, with the DN mutant-Gαi1 (murine) construct plus DN-Gαi3 construct (“DN-Gαi1/3”) or the vector control (“Vec”), were treated with SCF (50 ng/mL) for 5 min. The association of c-Kit, Grb2, Gab2, Shc, Gαi1, and Gαi3 was examined by co-immunoprecipitation (Co-IP) assays (A), and their expressions are shown in “Input” **(A);** The listed proteins in membrane fraction lysates and total cell lysates were examined **(B-D).** “Ctrl” refers to PBS treatment. *P < 0.05 versus “Vec”. ^#^*P* < 0.05 (B).

Next, the DN constructs were employed to prevent the association of Gαi1/3 with other proteins 26-28, 34. Specifically, DN-Gαi1 (murine) and the DN-Gαi3 (murine) constructs were co-transduced into WT MEFs, and after the selection of stable MEFs, “DN-Gαi1/3” MEFs, were obtained. In DN-Gαi1/3, the conserved Gly (G) residue was replaced by Thr (T) in the G3 box, to block the possible association of Gαi1/3 with other adaptor/associated proteins 30, 31. Co-IP assay results shown in Figure **2A** demonstrate that DN-Gαi1/3 disrupts SCF-induced c-Kit-Grb2-Gab2-Shc association but without affecting their expressions (Figure **2A**, “Input”). Only WT Gαi1 and Gαi3 but not the mutants were associated with c-Kit-Grb2-Gab2-Shc in response to SCF treatment (Figure **2A**). Expression of DN-Gαi1 and DN-Gαi3 was verified by Western blotting (Figure **2A**, “Input”). Importantly, SCF-induced c-Kit internalization, evidenced by a reduction in membrane c-Kit levels, was prevented by DN-Gαi1/3 (Figure **2B**). SCF-induced Gab2 and Shc phosphorylation was reduced in DN-Gαi1/3 (Figure **2C**). Consequently, SCF-induced Akt (Ser-473), S6K, and Erk1/2 phosphorylation were robustly suppressed in MEFs with DN-Gαi1/3 (Figure **2D**). Thus, DN-Gαi1/3 disrupted SCF-induced c-Kit internalization and adaptor protein binding and prevented downstream signaling.

### Gαi1 and Gαi3 silencing prevents SCF-induced signaling and pro-angiogenic activity in endothelial cells

**Figure 3:**
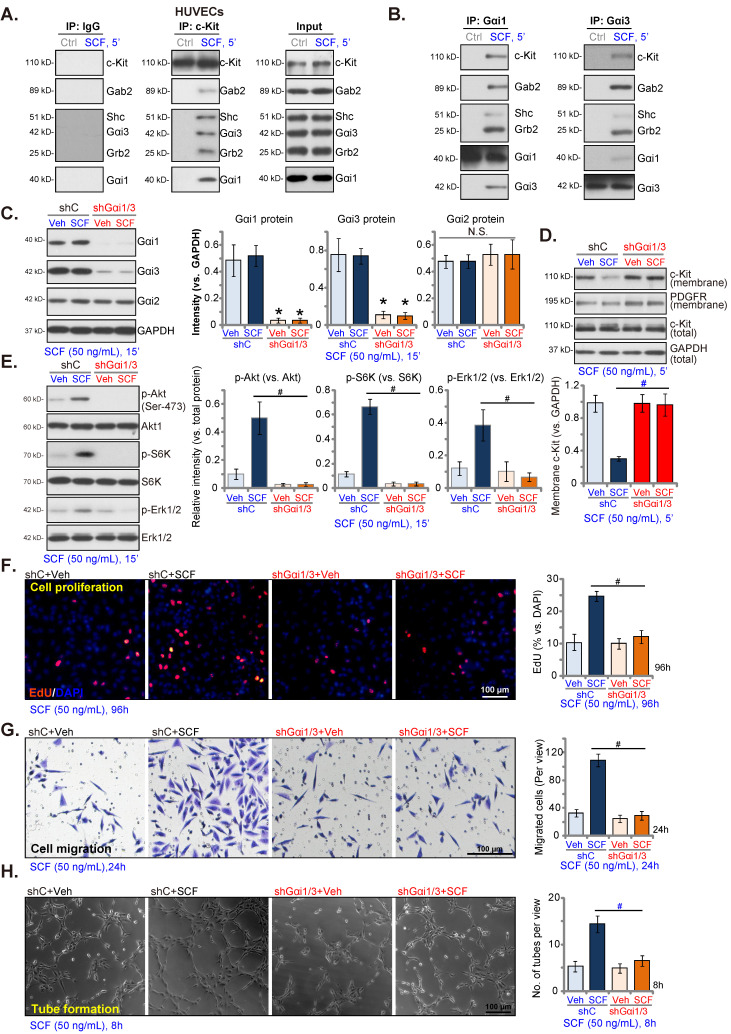
Gαi1 and Gαi3 silencing prevents SCF-induced signaling and pro-angiogenic activity in endothelial cells. HUVECs were treated with SCF (50 ng/mL) for 5 min, and the association of c-Kit, Grb2, Gab2, Shc, Gαi1, and Gαi3 was examined by co-immunoprecipitation (Co-IP) assays (**A** and **B**). Their expressions are shown as “Input” (**A** and **B**). Stable HUVECs, with the lentiviral human Gαi1 shRNA and the lentiviral human Gαi3 shRNA (“shGαi1/3”) or scramble shRNA control (“shC”), were treated with SCF (50 ng/mL) for 15 min, and the listed proteins in membrane fraction lysates and total cell lysates were examined (**C-E**); HUVECs were further cultured, and cell proliferation (EdU nuclear incorporation, **F**), *in vitro* migration (**G**), and capillary tube formation (**H**) were assessed. “Veh” refers to vehicle control. * ***P***< 0.05 versus “Veh” treatment in shC HUVECs.**^ #^*P*** < 0.05. Scale bar = 100 μm.

The roles of Gαi1 and Gαi3 in SCF-induced signaling in endothelial cells were studied. Co-IP assays were conducted and results showed that SCF-activated c-Kit immunoprecipitated with Grb2, Gab2, and Shc, as well as Gαi1 and Gαi3 in HUVECs (Figure **3A**). Expressions of c-Kit, Grb2, Gab2, Shc, Gαi1 and Gαi3 remained unchanged following SCF treatment (Figure **3A**, “Input”). Moreover, both Gαi1 and Gαi3 proteins formed a complex with c-Kit, Grb2, Gab2, and Shc in SCF-treated HUVECs (Figure **3B**). In HUVEC, SCF-induced cell proliferation (Figure **S3A**), migration (Figure **S3B**), and tube formation (Figure **S3C**), as well as the mRNA expression of *VEGF* (Figure **S3D**) and *PDGF-BB* (Figure **S3E**) were inhibited by the Erk1/2 inhibitor PD98059 or the PI3K-Akt-mTOR inhibitor LY294002. Importantly, PD98059 plus LY294002 (“PD+LY”) completely blocked SCF-induced pro-angiogenic actions along with the mRNA expression of *VEGF* and *PDGF-BB* in HUVECs (Figures **S3A**-**E**). Thus PI3K-Akt-mTOR and Erk are two essential cascades required for SCF-induced pro-angiogenic actions in HUVECs.

To knockdown Gαi1 and Gαi3 in endothelial cells, Gαi1 shRNA (human)- and the Gαi3 shRNA (human)-packed lentiviral particles, reported previously 25-27, 34, were transfected in HUVECs. After selection (by puromycin), stable HUVECs were established, namely “shGαi1/3” HUVECs. The protein expressions of Gαi1 and Gαi3 reduced remarkably in shGαi1/3 HUVECs (Figure **3C**), while that of Gαi2 remained unchanged (Figure **3C**) compared to HUVECs with lentiviral scramble non-sense shRNA control (“shC”). SCF (50 ng/mL, 15 min) single treatment failed to alter the expression of Gαi proteins in shC HUVECs (Figures **3B** and **C**).

Importantly, Gαi1 and Gαi3 silencing prevented SCF-induced membrane c-Kit internalization (Figure **3D**). Total c-Kit protein expression remained unchanged (Figure **3D**). Moreover, SCF-induced activation of downstream signaling was largely inhibited following Gαi1 plus Gαi3 knockdown in HUVECs (Figure **3E**). Akt (Ser-473), S6K, and Erk1/2 phosphorylation by SCF was almost blocked in shGαi1/3 HUVECs (Figure **3E**). Since Gαi1 and Gαi3 silencing blocked SCF-induced signaling in HUVECs, we next tested whether these affected cellular behaviors. In shC HUVECs, treatment with SCF (50 ng/mL) was pro-angiogenic, as evidenced by increased cell proliferation (Figure **3F**), *in vitro* migration (Figure **3G**), and capillary tube formation (Figure **3H**). After Gαi1 and Gαi3 silencing, SCF-induced pro-angiogenic activity was almost completely blocked (Figures **3F**-**H**). Thus, Gαi1/3 silencing prevented SCF-activated signaling and angiogenesis in cultured endothelial cells.

Further analyses showed that SCF-induced Gab2 phosphorylation in HUVECs was attenuated following Gαi1/3 shRNA (Figure **S4A**). Interestingly, Gab2 shRNA (“shGab2”) almost blocked SCF-activated Akt and Erk in HUVECs (Figure **S4B**), leaving Gαi1/3 and c-Kit protein expression unchanged (Figure **S4B**). Moreover, SCF-induced proliferation (Figure **S4C**), and migration (Figure **S4D**) of HUVECs were largely inhibited by Gab2 shRNA. These results support that Gαi1/3 are upstream proteins mediating Gab2 and downstream signaling activation in HUVECs, and are essential for SCF-induced angiogenesis in HUVECs.

### SCF-induced signaling and pro-angiogenic activity are inhibited by mutations in Gαi1 and Gαi3 in endothelial cells

**Figure 4:**
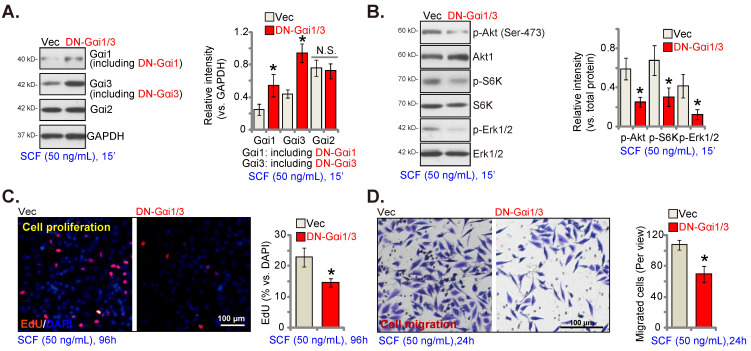
SCF-induced signaling and pro-angiogenic activity are inhibited in endothelial cells with Gαi1 and Gαi3 mutations. HUVECs, with the dominant negative (DN) mutant-Gαi1 (human) construct plus the DN Gαi3 (human) (“DN-Gαi1/3”), construct or the vector control (“Vec”), were treated with SCF (50 ng/mL) for 15 min, and expression of listed proteins is shown (**A** and **B**). HUVECs were further cultured, and cell proliferation (**C**) and *in vitro* migration (**D**) were tested. * ***P***< 0.05 versus “Vec” cells. “N. S.” denotes ***P*** > 0.05. Scale bar = 100 μm.

Next, DN mutant Gαi1 (human) and DN mutant Gαi3 (human) constructs were transduced into HUVECs. Stable HUVECs, namely “DN-Gαi1/3” HUVECs, were obtained after selection. The expression of the mutant Gαi proteins in DN-Gαi1/3 HUVECs was confirmed, and Gαi2 protein expression remained unchanged (Figure **4A**). SCF-induced phosphorylation (50 ng/mL) of Akt (Ser-473), S6K, and Erk1/2 was inhibited by DN-Gαi1/3 in HUVECs (Figure **4B**). Moreover, DN Gαi1/3 mutation robustly inhibited SCF-induced HUVEC proliferation (Figure **4C**) and *in vitro* migration (Figure **4D**).

### Gαi1 and Gαi3 overexpression strengthens SCF-induced signaling and pro-angiogenic activity in endothelial cells

**Figure 5:**
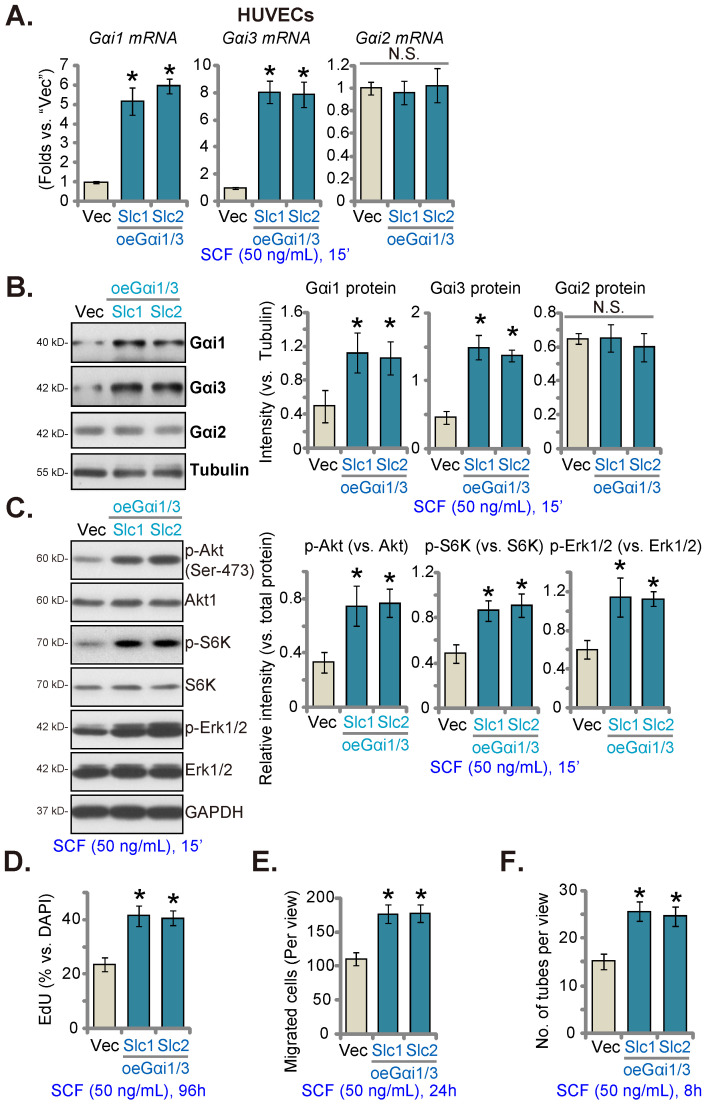
Gαi1 and Gαi3 overexpression strengthens SCF-induced signaling and pro-angiogenic activity in endothelial cells. HUVECs were transduced with the lentiviral human Gαi1-expressing construct plus the lentiviral human Gαi3-expressing vector, and two stable colonies, “oeGαi1/3-Slc1” and “oeGαi1/3-Slc2”, were obtained after selection. Control HUVECs were transduced with vector control (“Vec”). HUVECs were then treated with SCF (50 ng/mL) for 15 min and listed mRNA and protein levels were examined (**A-C**). HUVECs were further cultured, and cell proliferation (**D**), *in vitro* migration (**E**), and capillary tube formation (**F**) were tested. * ***P***< 0.05 versus “Vec”. “N. S.” denotes ***P*** > 0.05.

Since Gαi1/3 silencing, KO, or mutation largely inhibited SCF-induced signaling and pro-angiogenic activity in HUVECs, we next hypothesized that overexpressing Gαi1 and Gαi3 could exert opposite functions and augment pro-angiogenic activity in endothelial cells. Thus, the lentiviral particles with the Gαi1 (human)-expressing vector together with the lentiviral particles with the Gαi3 (human)-expressing vector were co-transfected into HUVECs, and puromycin was added to select two stable cell colonies, namely “oeGαi1/3-Slc1” and “oeGαi1/3-Slc2”. The expressions of Gαi1 and Gαi3 increased robustly in oeGαi1/3 HUVECs, while that of Gαi2 remained unchanged (Figures **5A** and **B**) compared to HUVECs with vector control (“Vec”). SCF-induced phosphorylation of Akt, S6K, and Erk1/2 was significantly augmented in oeGαi1/3-Slc1/2 HUVECs (Figure **5C**). Overexpressing Gαi1 and Gαi3 promoted HUVEC proliferation and increased EdU-positive nuclei ratio (Figure **5D**). Moreover, *in vitro* migration (Figure **5E**) and capillary tube formation (Figure **5F**) were strengthened in oeGαi1/3 HUVECs.

### Endothelial Gαi1/3 silencing prevents *in vivo* SCF-induced signaling and retinal angiogenesis

**Figure 6:**
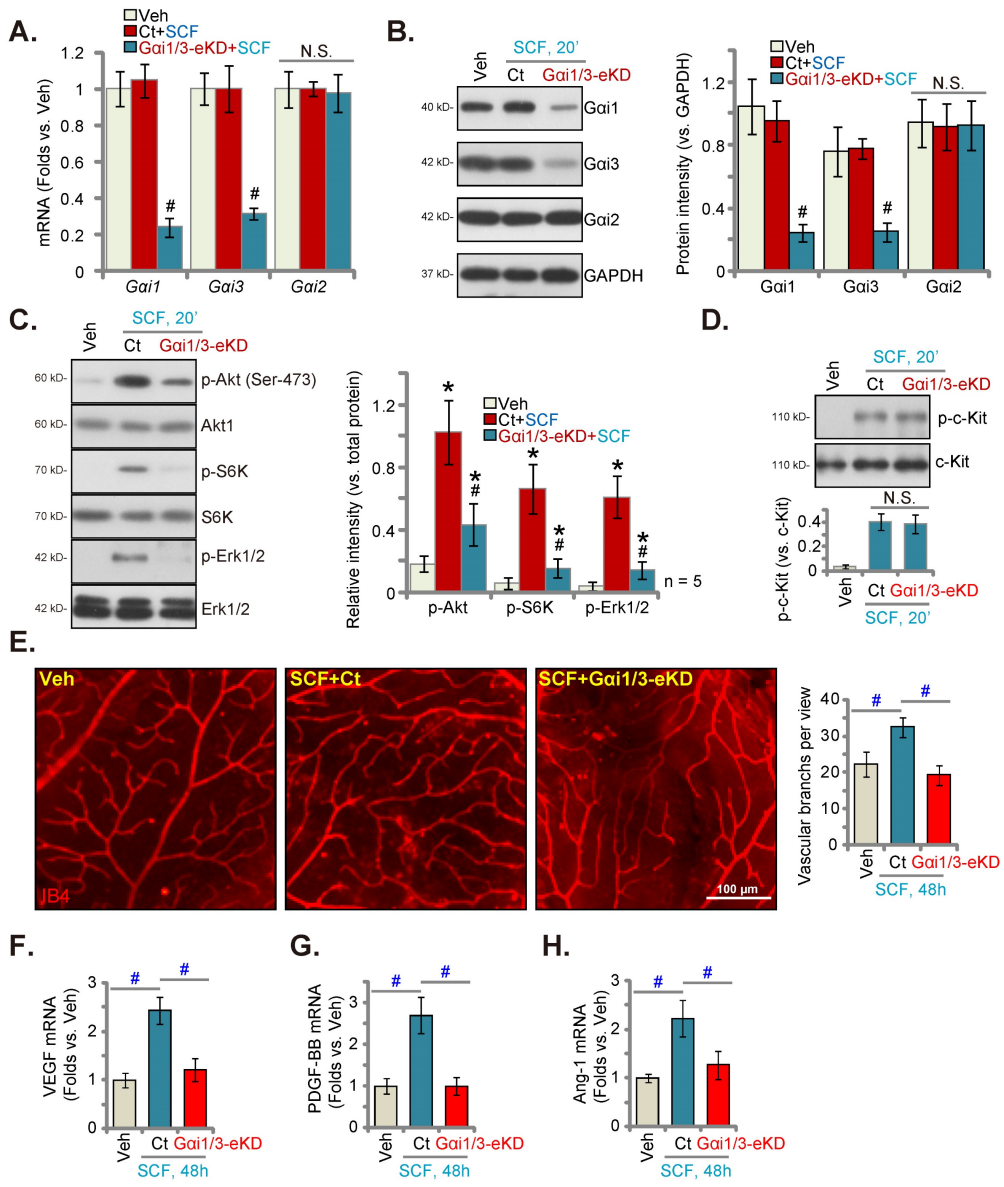
Endothelial Gαi1/3 silencing prevents SCF-induced signaling and retinal angiogenesis *in vivo*. One-month-old C57B/6 adult mice with AAV5-TIE1-Gαi1 shRNA plus AAV5-TIE1-Gαi3 shRNA (“Gαi1/3-eKD”) or the AAV5-TIE1-scramble shRNA control (“Ct”) were injected intravitreously with SCF (0.5 ng in 0.2 μL). After 20 min, the retinal tissues were collected and expressions of listed mRNAs and proteins in fresh tissues are shown (**A-D**). Alternatively, the retinal vasculature was visualized via IB4 staining after 48 h **(E).** The expressions of listed mRNAs are shown (**F-H**). * P < 0.05 versus vehicle control (“Veh”, saline) # P < 0.05 vs. “Ct” group. “N. S.” denotes P > 0.05. Scale bar = 100 μm.

Following a previously described protocol 23, 34, AAV5-TIE1-Gαi1 shRNA and AAV5-TIE1-Gαi3 shRNA were intravitreously injected to C57B/6 mice 34. This led to endothelial knockdown of Gαi1/3 (Gαi1/3-eKD 34) as the viral construct contained the binding sequence for the endothelial-specific promoter, TIE1 23, 34. The mRNA and protein expressions of Gαi1 and Gαi3 decreased robustly in the retinal tissues of the Gαi1/3-eKD mice (Figures 6A and B). Importantly, intravitreous injection of SCF enhanced Akt (Ser-473), S6K and Erk1/2 phosphorylation in the retinal tissues of vector control (“Ct”) mice (Figure 6C). SCF-activated signaling was substantially suppressed in the Gαi1/3-eKD mice (Figure 6C). Intravitreous SCF injection induced comparable c-Kit phosphorylation in retinal tissues of Ct and Gαi1/3-eKD mice (Figure 6D). The expression of total c-Kit remained unchanged (Figure 6D). Results of isolectin B4 (IB4) staining, shown in Figure 6E, demonstrated that SCF injection (for 48 h) enhanced the complexity of retinal vasculature, as evidenced by a dramatic increase in the number of vascular branches (and branch points) (Figure 6E). SCF-induced retinal angiogenesis was, however, remarkably inhibited in Gαi1/3-eKD mice (Figure 6E). mRNA expression of several important pro-angiogenic factors, including VEGF (Figure 6F), PDGF-BB (Figure 6G), and angiotensin-I (Ang-1) (Figure 6H), increased in SCF-injected mouse retinal tissues, which was largely inhibited following Gαi1/3-eKD (Figures 6F-H). Thus, endothelial Gαi1/3 knockdown prevented SCF-induced signaling and retinal angiogenesis *in vivo*.

### SCF shRNA inhibits pathological retinal angiogenesis in mice with diabetic retinopathy (DR)

**Figure 7:**
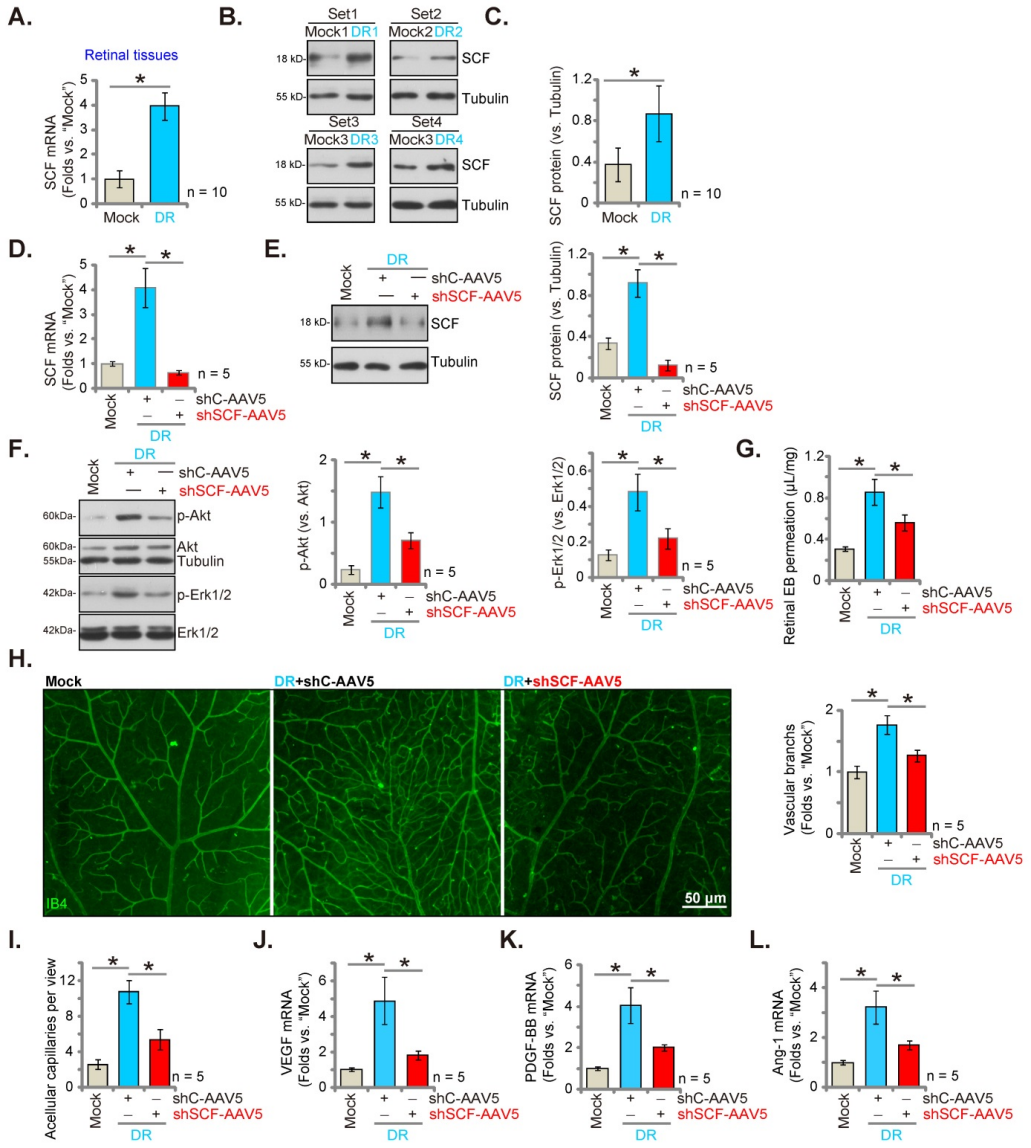
SCF shRNA inhibits pathological retinal angiogenesis in diabetic retinopathy (DR) mice. The retinal tissues of DR mice (90 days after the last STZ administration) and “mock” control mice (with citrate buffer administration) were separated, expressions of SCF mRNA and protein were tested, and the results were quantified (**A-C**). Day-30 after STZ administration, mice were injected intravitreously with AAV5-packed SCF shRNA (“shSCF-AAV5”, at 0.1 μL) or AAV5-packed scramble shRNA control (“shC-AAV5”, at 0.1 μL). After another 60 days, listed mRNAs and proteins in the retinal tissues were assessed (**D-F, J-L**). Alternatively, mice were infused with Evans blue (EB) for 2 h, and the percentage of EB leakage was quantified **(G).** IB4 staining was carried out to visualize the retinal vasculature (H, scale bar = 50 μm), and the average number of vascular branches were quantified (**H**). The retinal trypsin digestion assay was performed and the number of acellular capillaries per view were recorded (**I**). “Mock” refers to mice administered with citrate buffer. * P< 0.05.

We checked for alteration in the expression of SCF in streptozotocin (STZ)-administrated DR mice's retinal tissues. After 90 days of the last STZ administration, the retinal tissues of both DR and “Mock” control (citrate buffer-administrated) mice were collected. The mRNA expression of *SCF* in the retinal tissues of DR mice was significantly elevated (Figure **7A**). Moreover, protein upregulation of SCF was observed in the retinal tissues of a set of four representative STZ-administrated DR mice (Figure **7B**). After combining all 10 sets of blotting data, we found that the protein levels of *SCF* were significantly elevated in the retinal tissues of DR mice (Figure **7C**).

AAV5-SCF shRNA (“shSCF-AAV5”) or AAV5-scramble shRNA control (“shC-AAV5”) were injected intravitreously into the retina of DR mice on day-30 after the last STZ administration to examine whether increased SCF expression played a role in pathological retinal angiogenesis in DR mice. After another 60 days, the fresh retinal tissues were collected and examined. As shown, shSCF-AAV5 downregulated mRNA and protein expressions of *SCF* in shC-AAV5 DR mice's retinal tissues (Figures **7D** and **E**). Akt-Erk1/2 phosphorylation increased in shC-AAV5 DR mice's retinal tissues (Figure **7F**). Remarkably, SCF silencing by shSCF-AAV5 reduced Akt and Erk activation in DR mice's retinal tissues (Figure **7F**).

The retinal vascular leakage, tested by Evans blue (EB) quantification, increased significantly in shC-AAV5 DR mice compared to the mock control mice (Figure **7G**). IB4 staining assay results revealed enhanced retinal vasculature complexity with increased vascular branches and branch points in the retina of shC-AAV DR mice, further supporting retinal pathological angiogenesis (Figure **7H**). Retinal trypsin digestion assay showed an increase number of retinal acellular capillaries in shC-AAV DR mice (Figure **7I**).

Importantly, SCF silencing, through intravitreous injection of shSCF-AAV5, largely inhibited pathological retinal angiogenesis in DR mice. Specifically, in the DR mice retinal vascular leakage (Figure **7G**), pathological angiogenesis (Figure **7H**), and acellular capillary formation (Figure **7I**) were largely suppressed by SCF silencing through shSCF-AAV5. Thus, SCF silencing ameliorated pathological retinal angiogenesis in DR mice. mRNA expressions of *VEGF* (Figure **7J**), *PDGF-BB* (Figure **7K**), and *Ang-1* (Figure **7L**) increased substantially in the retinal tissues of DR mice, which was suppressed by shSCF-AAV5 injection (Figures **7J**-**L**).

### SCF shRNA ameliorates degeneration of retinal ganglion cells (RGCs) in DR mice

**Figure 8:**
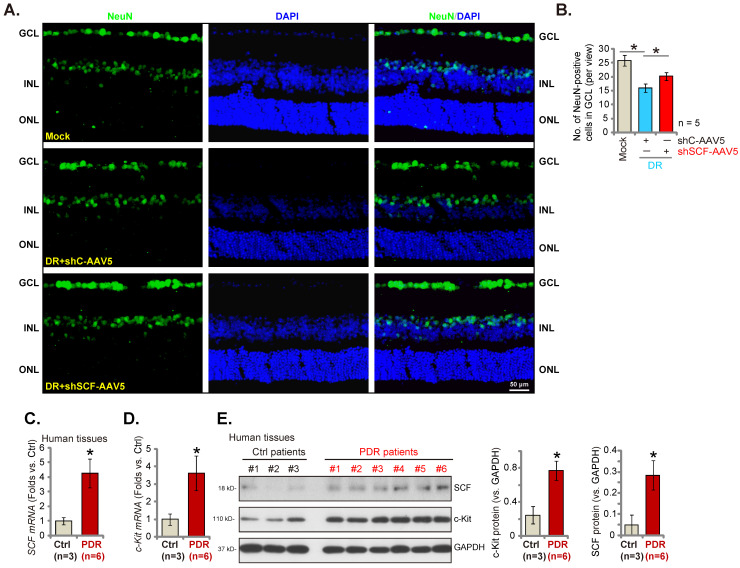
SCF shRNA ameliorates degeneration of retinal ganglion cells (RGCs) in diabetic retinopathy (DR) mice. Day-30 after STZ administration, mice were injected intravitreously with AAV5-packed SCF shRNA (“shSCF-AAV5”, at 0.1 μL) or AAV5-packed scramble shRNA control (“shC-AAV5”, at 0.1 μL). After another 60 days, NeuN-positive RGCs in GCL were detected (**A** and **B**, scale bar = 50 μm). The listed human tissues were homogenized and mRNA and protein expressions of SCF and c-Kit were examined (C-E). “Mock” refers to mice administered with citrate buffer. “GCL” is the ganglion cell layer; “ONL” is the outer nuclear layer; “INL” is the inner nuclear layer. * P< 0.05 (A and B). * P< 0.05 vs. “Ctrl” tissues (**C-E**).

In the pathogenesis of DR, pathological angiogenesis, energy crisis, oxidative injury, and inflammatory reaction, all lead to the degeneration of RGCs and are important mechanisms causing blindness 36, 37. The number of NeuN-stained RGCs in GCL (ganglion cell layer) decreased substantially in the retina of shC-AAV DR mice compared to the mock control mice (Figures **8A** and **B**). Importantly, SCF shRNA by intravitreous injection of AAV5-SCF shRNA largely inhibited RGC degeneration in DR mice (Figures **8A** and **B**).

### SCF and c-Kit expressions increase significantly in proliferative retinal tissues of human patients with proliferative diabetic retinopathy (PDR)

Lastly, the expressions of SCF and c-Kit in human patients' proliferative retinal tissues were tested. We evaluated the previously-described human tissue samples 23, 27. Retinal proliferative membrane tissues of six different human PDR patients along with the retinal tissues of three age-matched traumatic retinectomy patients were obtained 23, 27. The mRNA (Figures **8C**and **D**) and protein (Figure **8E**) expressions of *SCF* and *c-Kit* increased substantially in human PDR patients' proliferative retinal tissues.

## Discussion

Akt-mTOR and Erk cascade activation are vital for SCF/c-Kit-induced HUVEC survival, migration, and capillary tube formation *in vitro* and angiogenesis* in vivo* 18, 38. Herein, we discovered that Gαi1/3 are essential proteins mediating SCF-activated signaling and angiogenesis. In MEFs and HUVECs, SCF-induced Akt-mTOR and Erk activation was prevented by Gαi1/3 silencing, KO, or DN mutations but was strengthened following ectopic overexpression of Gαi1/3. SCF-stimulated HUVEC proliferation, migration, and capillary tube formation were substantially suppressed after Gαi1/3 shRNA, KO, or DN mutations but were greatly enhanced following Gαi1/3 overexpression. *In vivo*, Gαi1 and Gαi3 endothelial knockdown potently reduced SCF-induced Akt-mTOR and Erk activation in retinal tissues and retinal angiogenesis in mice.

Following SCF stimulation, the Grb2-Sos complex recruitment to c-Kit was through the association with tyrosine-phosphorylated Shc, which mediated Src family kinase (SFK) phosphorylation and activated Erk-MAPK signaling downstream 39. PI3K could be activated by SCF by binding to Gab2 40, 41. Herein, Gαi1/3 was associated with SCF-activated c-Kit in MEFs and HUVECs, which was essential for binding and activation of key adaptor proteins (Grb2, Gab2, and Shc) and transducing signals downstream. Gαi1/3 DN mutation disrupted SCF-induced binding of adaptor proteins to c-Kit and prevented Akt-mTOR and Erk activation downstream.

Nishida *et al.,* reported that Gab2 was tyrosine phosphorylated in response to SCF stimulation 42. SCF-activated Akt and MAPK activation was largely impaired in bone marrow-derived mast cells with Gab2 KO 42. Sun *et al.,* supported a role of Gab2 in mediating PI3K activation by SCF-activated c-Kit 43. Following SCF stimulation phosphorylated Gab2 associated with c-Kit and Shp-2, required for downstream signaling transduction 43, 44. Here we found that SCF-induced c-Kit-Gab2 association and Gab2 phosphorylation were largely inhibited by Gαi1/3 depletion or DN mutations. Thus Gαi1/3 shall act as upstream proteins mediating SCF-induced Gab2 activation.

With SCF binding, the receptor c-Kit clusters as dimers and internalizes by endocytosis possibly in clathrin-coated pits 45, 46. Like other RTKs, SCF-activated c-Kit internalization is a controlled process assembled by the endocytic machinery including clathrin chains, adaptor proteins, dynamin, and other cytosolic factors 45, 46. This process is important for binding to adaptor proteins, downstream signaling activation, and receptor recycling 45-48.

Our previous findings indicate that Gαi1/3 binding to ligand-activated receptors is essential for receptor internalization and endocytosis 27, 28, 33, 35. Gαi1/3's association with VEGF-stimulated VEGFR2 to initiate VEGFR2 endocytosis was essential for the downstream signaling activation 27. Gαi1/3 immunoprecipitated with IL-4-stimulated IL-4Rα at the intracellular domain in macrophages, thus promoting endosomal traffic of IL-4Rα and activating the Akt-mTOR cascade downstream 33. In lipopolysaccharide (LPS)-stimulated macrophages, Gαi1/3 binding to CD14 promoted toll-like receptor 4 (TLR4) endocytosis, Gab1 association, and downstream signaling 35. Gαi1/3 depletion or mutations prevented BDNF-induced TrkB endocytosis in neurons, thereby repressing the downstream signaling 28. Herein, we found that Gαi1/3's association with SCF-activated c-Kit was required for membrane c-Kit internalization and endocytosis in MEFs and HUVECs. Gαi1/3 silencing, KO, or DN mutations prevented SCF-induced c-Kit endocytosis. This could be a key mechanism underlying Gαi1/3-mediated SCF/c-Kit signaling transduction.

Hypoxia upregulates c-Kit in endothelial cells, leading to remarkably enhanced angiogenic responses to SCF 49. In mouse ocular neovascularization models, expressions of c-Kit and SCF are markedly enhanced in ocular tissues 49. Conversely, blockade of the SCF/c-Kit cascade in *c-Kit* mutant mice or using anti-SCF antibody remarkably ameliorates pathological ocular neovascularization 49. Our results demonstrated that expressions of SCF and c-Kit increased significantly in human PDR patients' proliferative retinal tissues. mRNA and protein expressions of *SCF* increased dramatically in STZ DR mice retinal tissues. Importantly, SCF silencing, through intravitreous injection of SCF shRNA AAV, inhibited pathological retinal angiogenesis and RGC degeneration in DR mice. In addition to our previous findings showing Gαi1/3 upregulation in PDR patients' proliferative retinal tissues 27, we propose that augmented SCF-c-Kit-Gαi1/3 cascade is vital for the pathological angiogenesis in PDR, representing a promising therapeutic target against PDR and other ocular neovascularization diseases.

In DR, pathological angiogenesis in retinas will lead to severe ischemia and hypoxia environment, causing glutamate toxicity, oxidative injury and inflammation 36, 37, 50. These events will eventually lead to degeneration of RGCs and vision loss 36, 37, 50. Here *SCF* mRNA and protein expression was robustly increased in STZ DR mice retinal tissues. SCF silencing, through intravitreous injection of SCF shRNA AAV, potently suppressed pathological retinal angiogenesis and restored RGCs. Thus, in DR retinas, SCF silencing-induced amelioration of pathological angiogenesis should be far more powerful in restoring RGCs than possible decreased SCF-mediated direct neuroprotection 51.

## Material and Methods

### Reagents

Polybrene, SCF, puromycin, and serum were purchased from Sigma-Aldrich (St. Louis, MO). Antibodies were obtained from Santa Cruz Biotech (Santa Cruz, CA), Cell Signaling Technology (Beverly, MA), and Abcam (Shanghai, China). Other reagents were procured from Gibco-BRL (Suzhou, China).

### Cells

The previous studies describe different MEF lines 26-28, 30, 31, 34. The primary culture of HUVECs has also been reported before 23, 27, 52.

### Genetic modifications of Gαi1/3

As described previously 25, 27, 28, 34, genetic modifications in Gαi1/3's expression and function were achieved through different viral constructs. These modifications, including Gαi1/3 silencing by targeted shRNA, ectopic overexpression, and DN mutations, were performed in both MEFs and HUVECs 27, 28, 34. The puromycin-containing complete medium was used to establish stable cell lines.

### Other assays

Cellular functional assays, including the EdU test for cell proliferation, cell migration, and *in vitro* capillary tube formation, have been described previously 23, 27, 52. Protocols for western blotting, qRT-PCR, and Co-IP assays were described in the previous studies 31, 53, 54. We followed a previously described protocol for the isolation of cell plasma membrane 55 with minor modifications 28. All the primers and viral constructs were synthesized by Genechem (Shanghai, China).

### Human tissues

The human tissues used herein have been described previously 23, 27 The protocols were approved by the Ethics Committee of Soochow University (#BR-2019-012).

### STZ injection and DR mice

C57BL/6 mice (weighing 23.5-25.2 g and 6-8-week-old) were made to fast and intraperitoneally (*i.p.*) injected with STZ, 60 mg/kg, daily for 5 days. Mice with blood glucose levels over 300 mg/dL were considered diabetic. Age-matched control mice (“mock”) were injected with citrate buffer. The isolectin B4 (IB4) staining of retinal vasculature, retinal vascular leakage assay by Evans blue (EB) staining, retinal trypsin digestion assay for acellular capillary formation, retinal NeuN immunofluorescence staining, and hematoxylin-eosin (HE) staining were performed following previously reported protocols 23, 34. The protocols were approved by the Ethics Committee of Soochow University (#BR-2019-012).

### Intravitreal injection of AAV and retinal vasculature detection

The adult C57BL/6 mice, (23.5-25.2 g, 6-8-week-old) were anesthetized, and intravitreal injection of the virus was performed as reported previously 23, 34. Gαi1/Gαi3 shRNA sequences were inserted into an adeno-associated virus 5 (AAV5)-TIE1 construct 23 containing the sequence of the endothelial-specific promoter, TIE1. AAV injection (0.1 μL virus per mouse) was performed following a previously reported protocol 23.

### Statistical analysis

All data are presented as mean ± standard deviation (SD). All *in vitro* cell experiments and *in vivo* animal experiments were repeated at least five times. The blot data or qRT-PCR data quantifications were based on five replicate experiments unless otherwise stated. Statistical differences were calculated by Student's t-test (comparing two groups) or by one-way analysis of variance (ANOVA) followed by Dunnett's post hoc test. P< 0.05 was considered statistically significant.

## Supplementary Material

Supplementary figures.
