# Supplementary material for: SCF/c-Kit-activated signaling and angiogenesis require Gαi1 and Gαi3
Source: Int J Biol Sci. 2023 Mar 27;19(6):1910–24. doi: 10.7150/ijbs.82855 (PMC10092767; doi:10.7150/ijbs.82855)

Figure S1

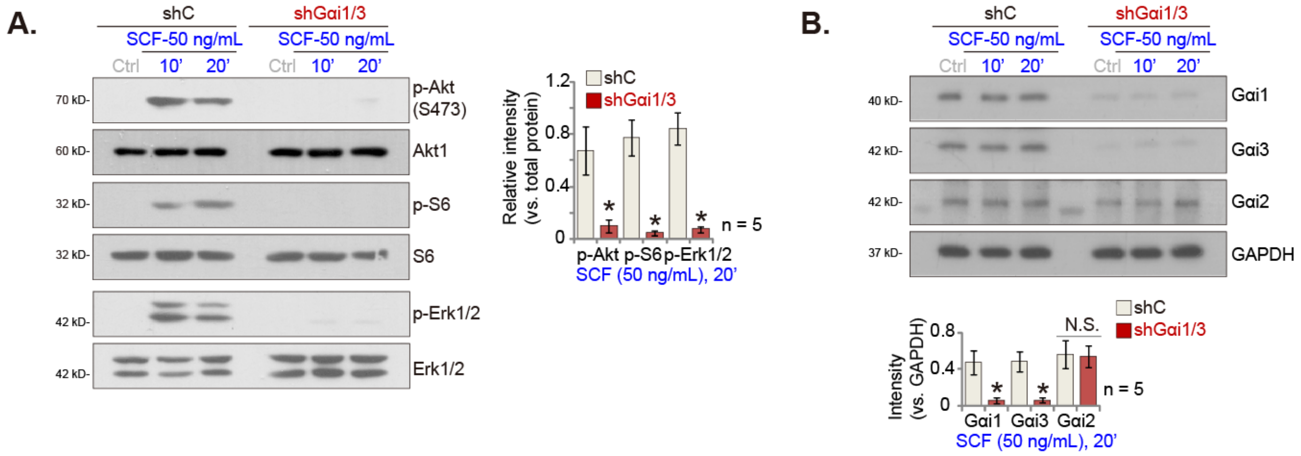

Figure S1. Gαi1 and Gαi3 are required for SCF-induced Akt-mTOR and Erk activation in MEFs. WT MEFs, with the lentiviral Gαi1 (murine) shRNA plus the lentiviral Gαi3 (murine) shRNA ("shGαi1/3") (**A**-**B**) or the scramble control shRNA ("shC") (**A**-**B**), were cultivated and treated with SCF (50 ng/mL) for designated time, listed proteins were tested (**A**-**B**); Gαi1/2/3 protein expression and protein phosphorylation were quantified (**A**-**B**). "Ctrl" stands for PBS treatment. ****P*** < 0.05 versus "shC". "N. S." stands for ***P*** > 0.05.

Figure S2

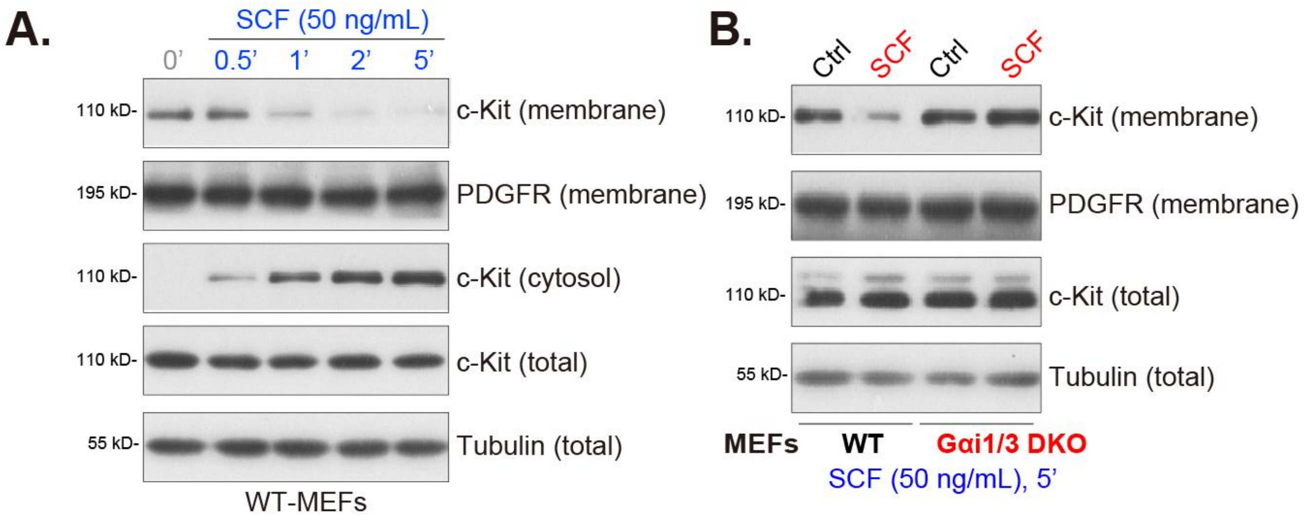

Figure S2. Gαi1 and Gαi3 are important for SCF-induced membrane c-Kit internalization in MEFs. WT MEFs were treated with SCF (50 ng/mL) for 0.5-5 min, listed proteins in membrane fraction lysates, cytosol fraction lysates and total cell lysates were examined (**A**). WT or Gαi1/3 DKO MEFs were treated with SCF (50 ng/mL) for 5 min, listed proteins in membrane fraction lysates and total cell lysates were examined (**B**). “Ctrl” stands for PBS treatment.

Figure S3.

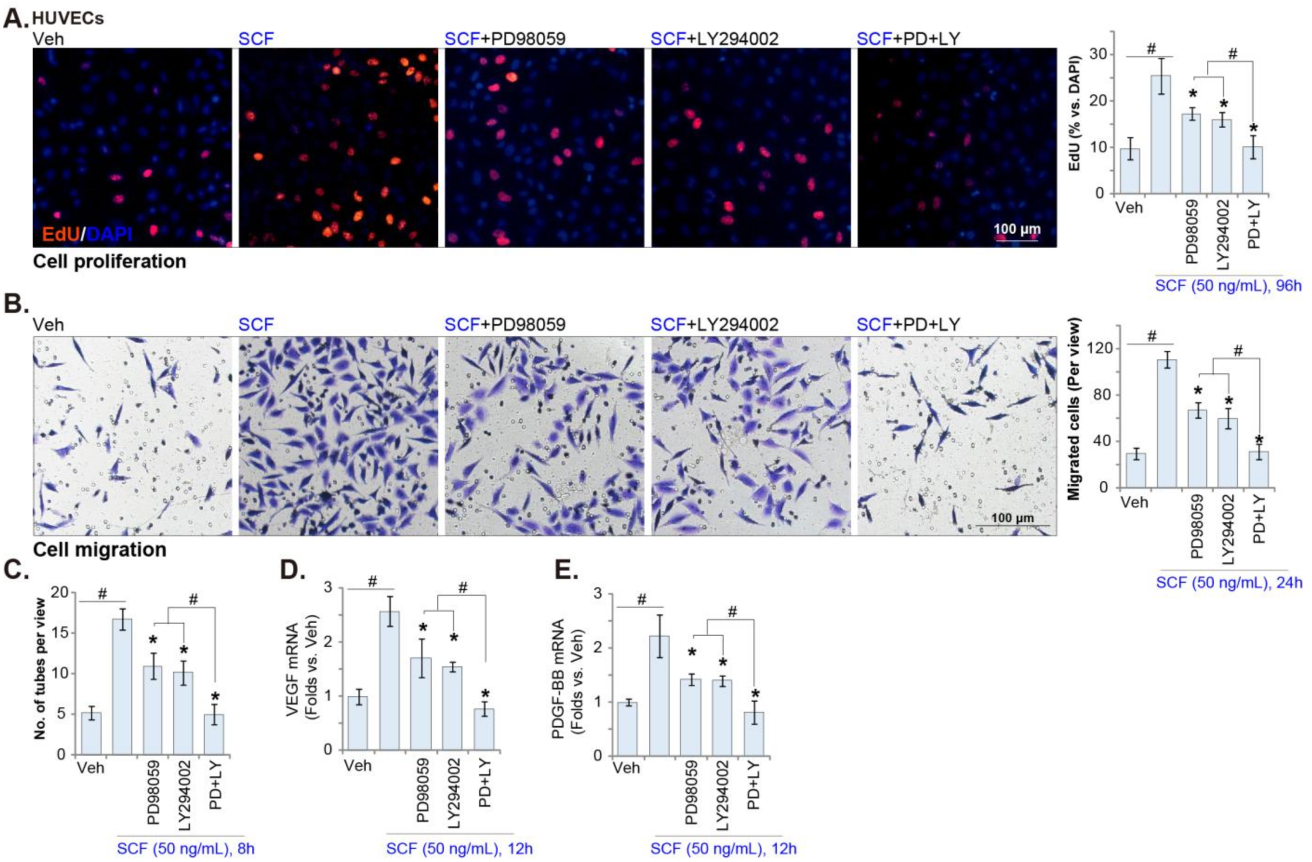

Figure S3. PI3K-Akt-mTOR and Erk inhibition suppresses SCF-induced pro-angiogenic activity in HUVECs. HUVECs were pretreated with PD98059 (5 μM), LY294002 (5 μM) or PD98059 plus LY294002 ("PD+LY") for 30 min, followed by SCF (50 ng/mL) treatment; HUVECs were cultivated for applied time periods, cell proliferation (**A**), migration (**B**) and formed tubes (**C**) were tested. Expression of listed mRNAs was examined (**D** and **E**). "Veh" stands for vehicle control group. * ***P***< 0.05 versus "SCF" only treatment. $^{\#}$***P***< 0.05. Scale bar = 100 μm.

Figure S4.

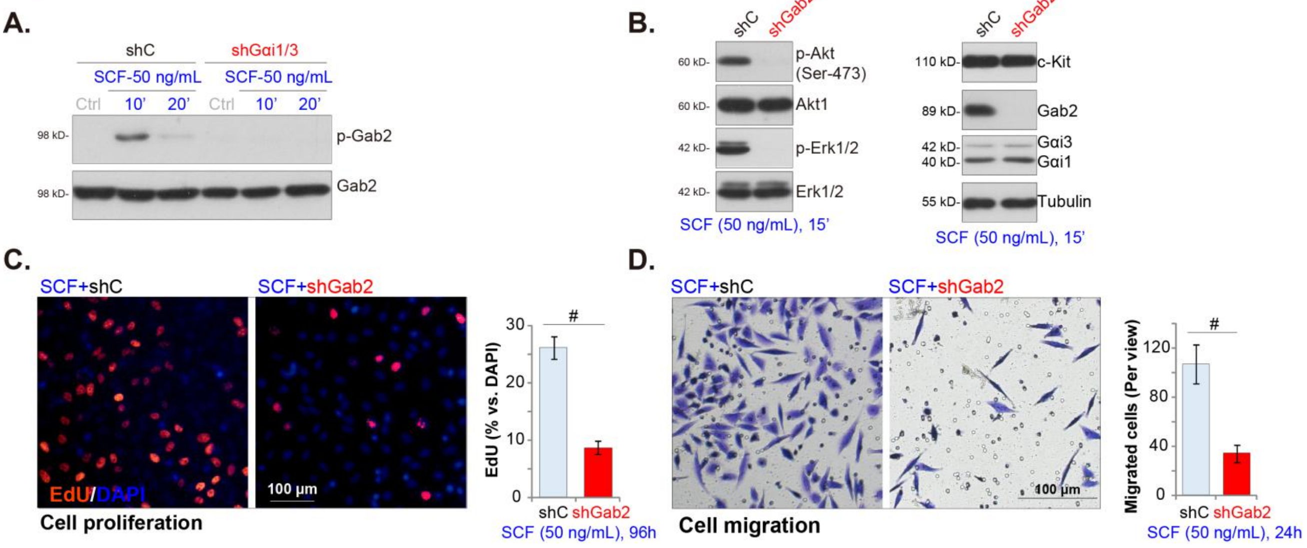

Figure S4. Gab2 is vital for SCF-induced signaling and angiogenesis in HUVECs. Stable HUVECs, with the lentiviral human Gαi1 shRNA plus lentiviral human Gαi3 shRNA ("shGαi1/3") or scramble control shRNA ("shC"), were treated with SCF (50 ng/mL) for 10/20 min, listed proteins were examined (**A**). Stable HUVECs, with the lentiviral Gab2 shRNA ("shGab2") or scramble control shRNA ("shC"), were treated with SCF (50 ng/mL) for 15 min, listed proteins were examined (**B**); HUVECs were further cultivated for applied time, cell proliferation (**C**) and *in vitro* migration (**D**) were examined. $^{\#}\boldsymbol{P} < 0.05$. Scale bar = 100 μm.

Figure S5. The uncropped blotting images of the study.

Figure 1

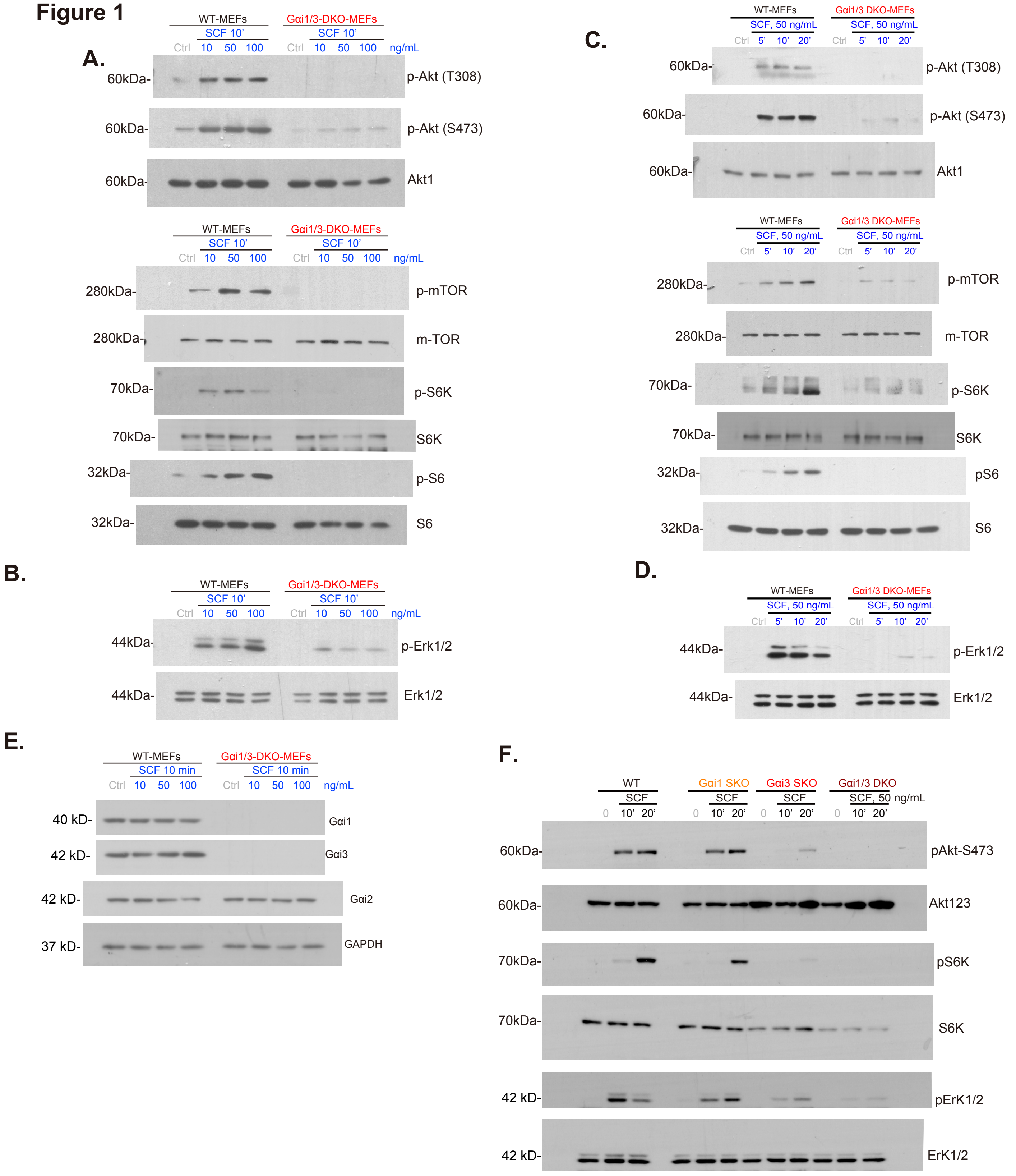

Figure 2

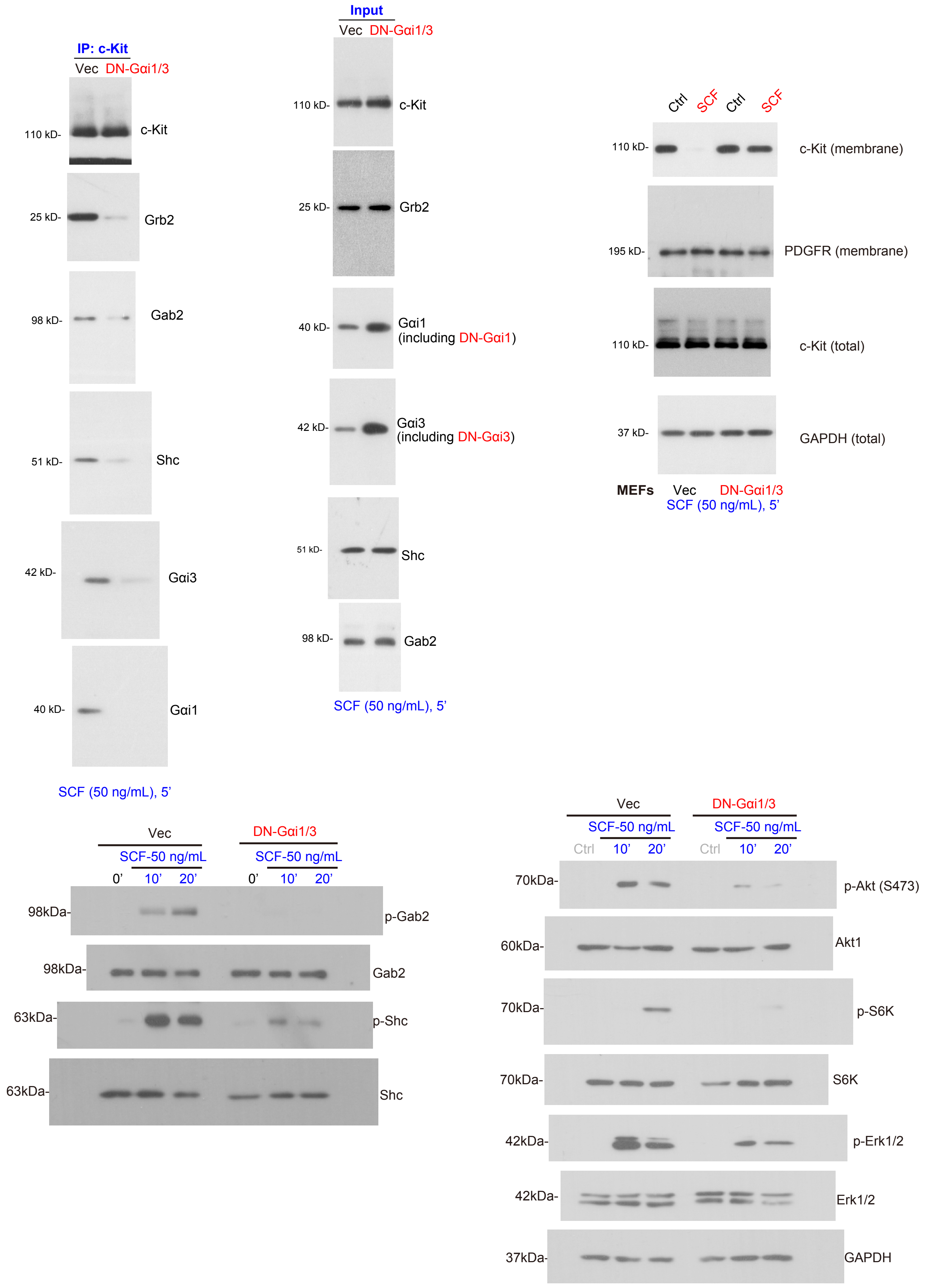

IP: c-Kit
Vec DN-Gαi1/3
110 kD- c-Kit
25 kD- Grb2
98 kD- Gab2
51 kD- Shc
42 kD- Gαi3
40 kD- Gαi1
SCF (50 ng/mL), 5'
Input
Vec DN-Gαi1/3
110 kD- c-Kit
25 kD- Grb2
40 kD- Gαi1 (including DN-Gαi1)
42 kD- Gαi3 (including DN-Gαi3)
51 kD- Shc
98 kD- Gab2
SCF (50 ng/mL), 5'
Ctrl SCF Ctrl SCF
110 kD- c-Kit (membrane)
195 kD- PDGFR (membrane)
110 kD- c-Kit (total)
37 kD- GAPDH (total)
MEFs Vec DN-Gαi1/3
SCF (50 ng/mL), 5'
Vec DN-Gαi1/3
SCF-50 ng/mL SCF-50 ng/mL
0' 10' 20' 0' 10' 20'
98kDa- p-Gab2
98kDa- Gab2
63kDa- p-Shc
63kDa- Shc
Vec DN-Gαi1/3
SCF-50 ng/mL SCF-50 ng/mL
Ctrl 10' 20' Ctrl 10' 20'
70kDa- p-Akt (S473)
60kDa- Akt1
70kDa- p-S6K
70kDa- S6K
42kDa- p-Erk1/2
42kDa- Erk1/2
37kDa- GAPDH

Figure 3.

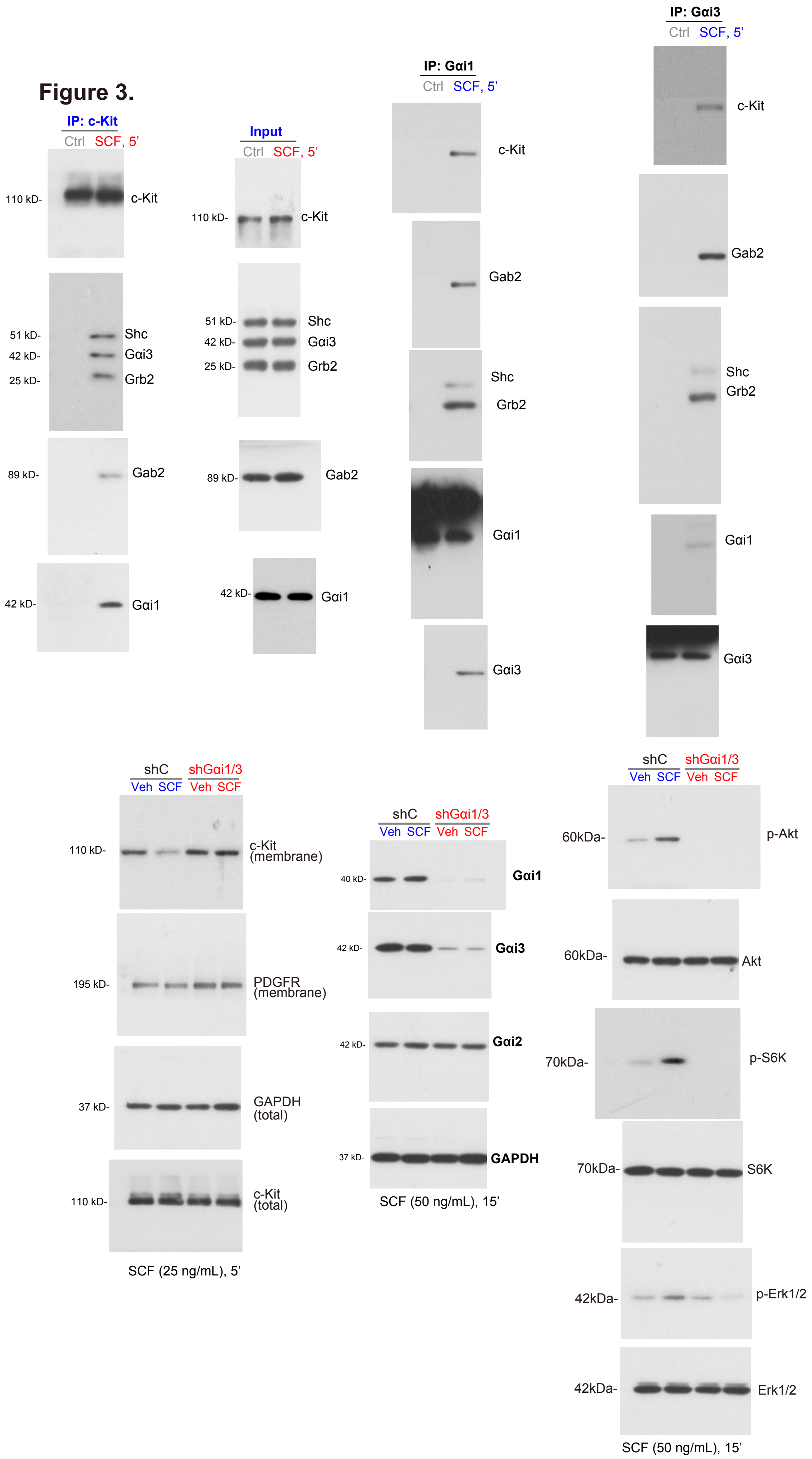
IP: c-Kit
Ctrl SCF, 5'
110 kD-
c-Kit
51 kD-
42 kD-
25 kD-
Shc
Gαi3
Grb2
89 kD-
Gab2
42 kD-
Gαi1
Input
Ctrl SCF, 5'
110 kD-
c-Kit
51 kD-
42 kD-
25 kD-
Shc
Gαi3
Grb2
89 kD-
Gab2
42 kD-
Gαi1
IP: Gαi1
Ctrl SCF, 5'
c-Kit
Gab2
Shc
Grb2
Gαi1
Gαi3
IP: Gαi3
Ctrl SCF, 5'
c-Kit
Gab2
Shc
Grb2
Gαi1
Gαi3
shC shGαi1/3
Veh SCF Veh SCF
110 kD-
c-Kit (membrane)
195 kD-
PDGFR (membrane)
37 kD-
GAPDH (total)
110 kD-
c-Kit (total)
SCF (25 ng/mL), 5'
shC shGαi1/3
Veh SCF Veh SCF
40 kD-
Gαi1
42 kD-
Gαi3
42 kD-
Gαi2
37 kD-
GAPDH
SCF (50 ng/mL), 15'
shC shGαi1/3
Veh SCF Veh SCF
60kDa-
p-Akt
60kDa-
Akt
70kDa-
p-S6K
70kDa-
S6K
42kDa-
p-Erk1/2
42kDa-
Erk1/2
SCF (50 ng/mL), 15'

Figure 4.

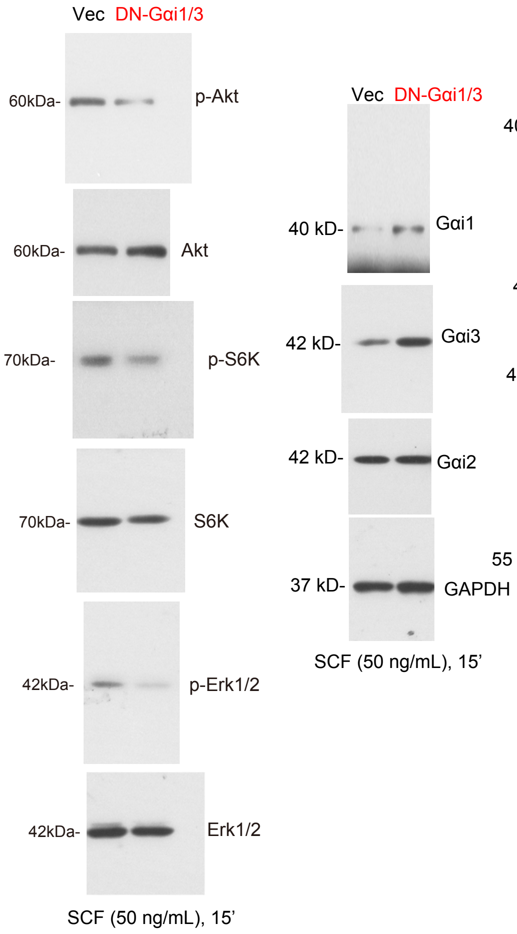

Figure 5.

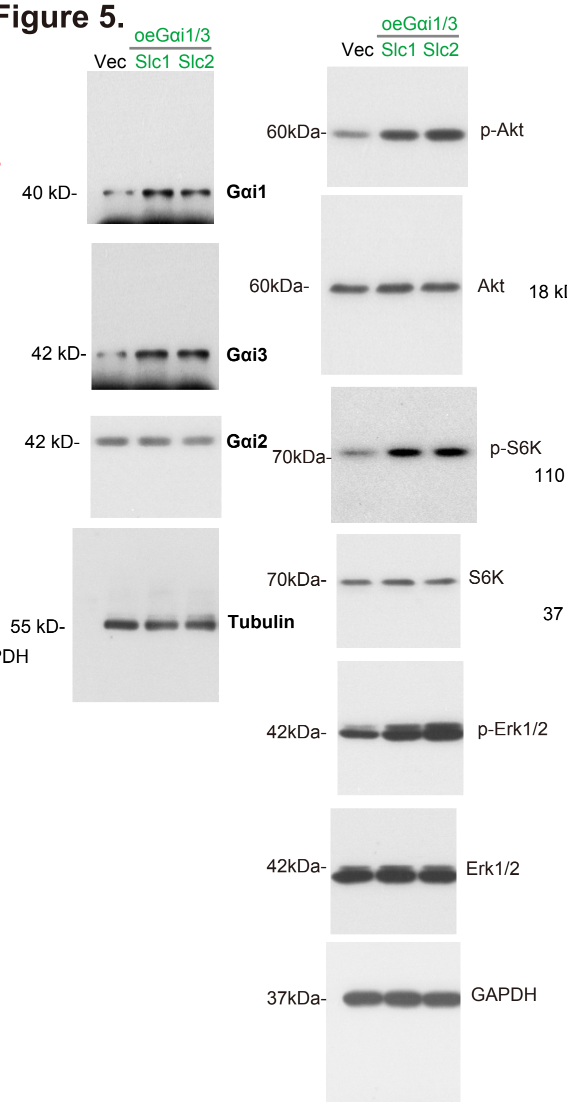

Figure 8.

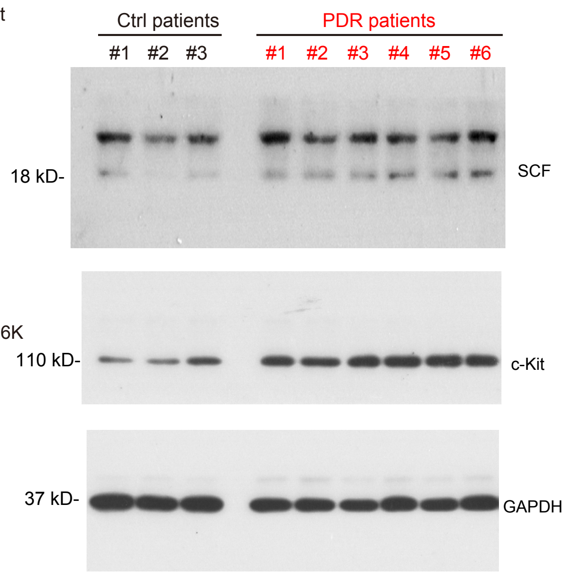

Figure 6

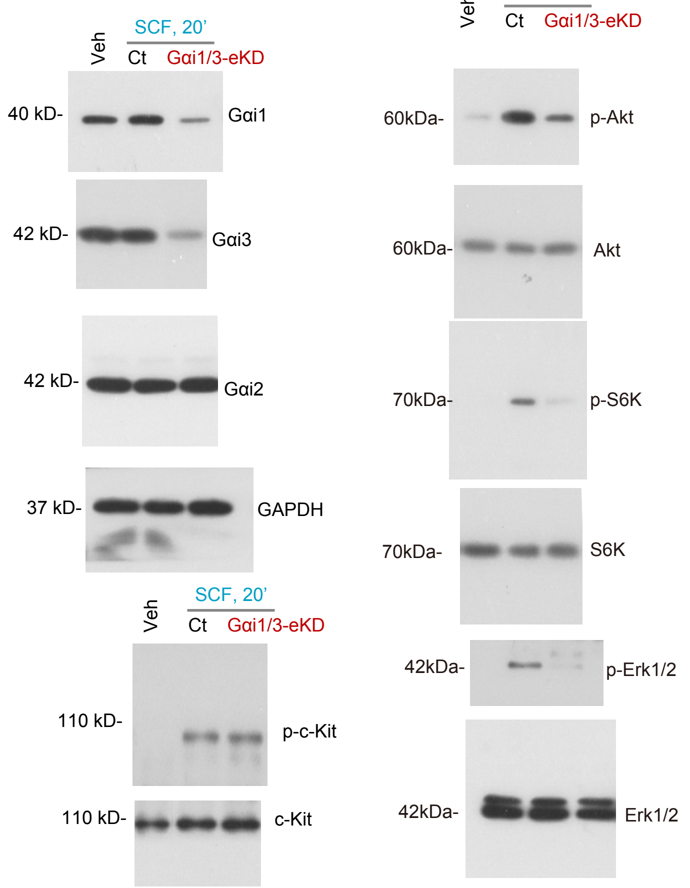

Figure 7.

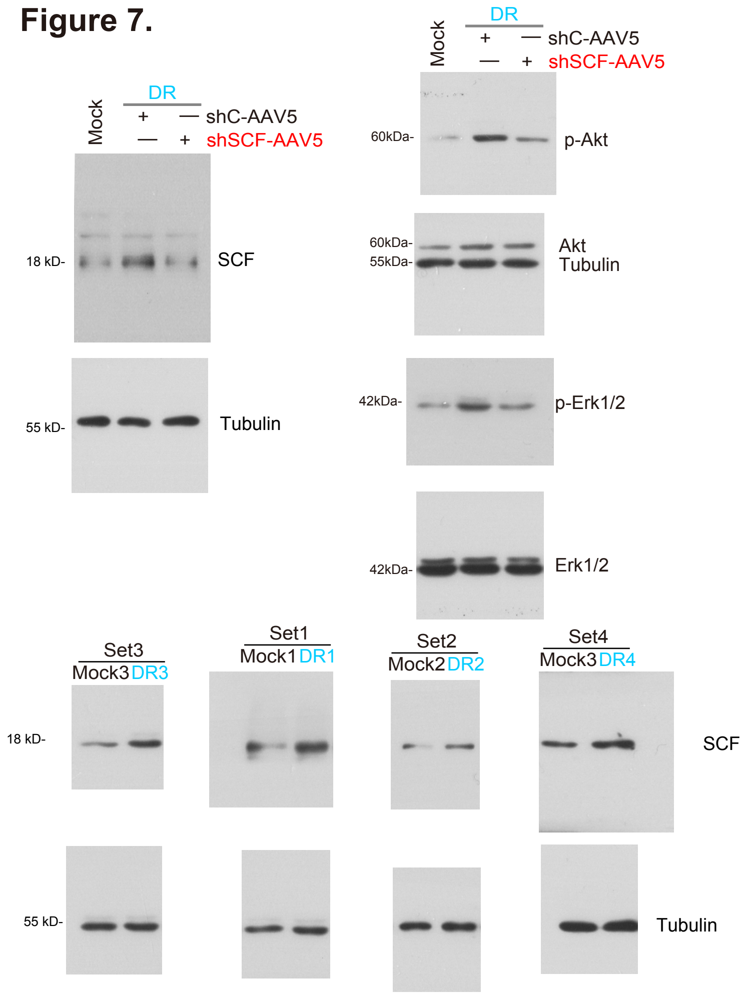

Figure S1

A.

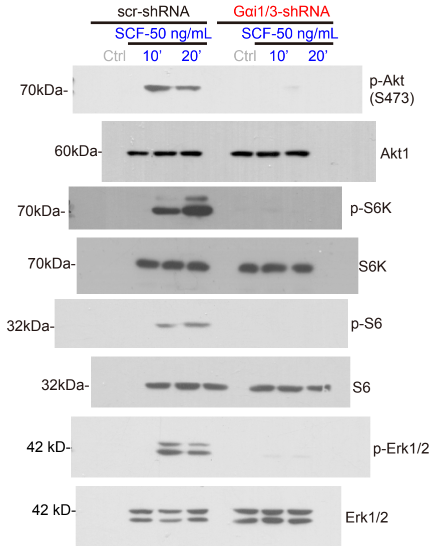

B.

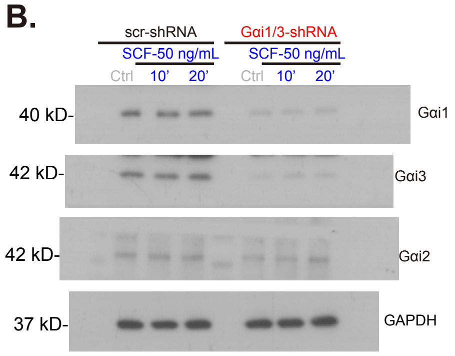

Figure S2

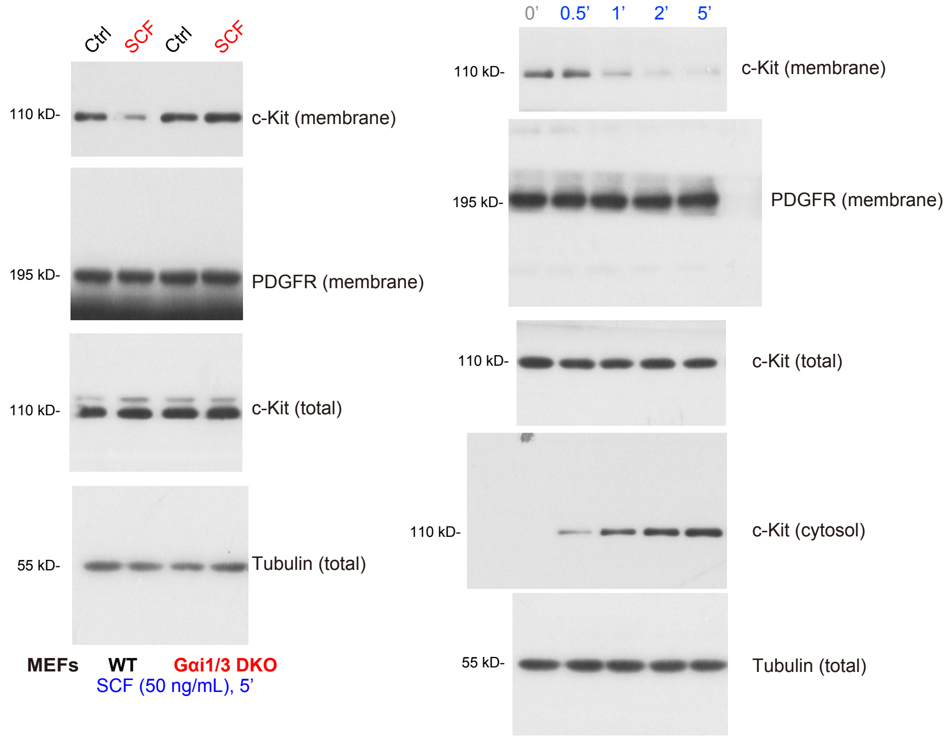

Figure S4.

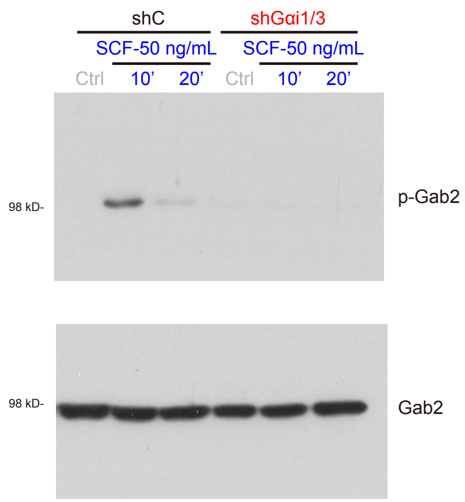

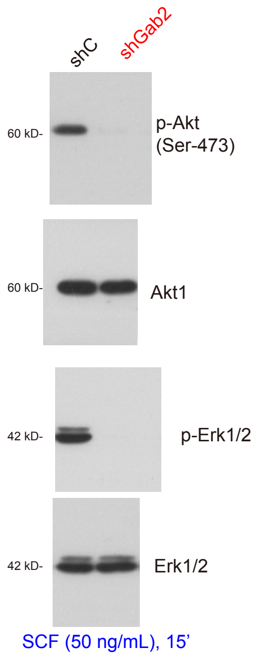

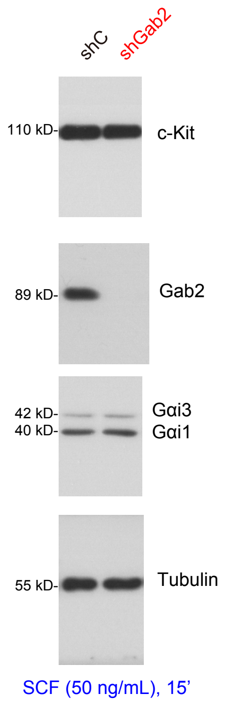

Supplement: Supplementary file 1 — Supplementary figures. [file ijbsv19p1910s1.pdf]
